# Using HbA1c measurements and the Finnish Diabetes Risk Score to identify undiagnosed individuals and those at risk of diabetes in primary care

**DOI:** 10.1186/s12889-023-15122-y

**Published:** 2023-01-31

**Authors:** Elín Arnardóttir, Árún K. Sigurðardóttir, Marit Graue, Beate-Christin Hope Kolltveit, Timothy Skinner

**Affiliations:** 1 School of Health Sciences University of Akureyri, Akureyri, Iceland; 2 Health Care Institution of North Iceland in Siglufjordur, Siglufjordur, Iceland; 3 Akureyri Hospital, Akureyri, Iceland; 4 Department of Health and Caring Sciences, Western Norway University of Applied Sciences, Bergen, Norway; 5 Institute of Psychology, University of Copenhagen, Copenhagen, Denmark; 6 Australian Centre for Behavioural Research in Diabetes, Melbourne, Victoria Australia

**Keywords:** Prediabetes, Screening, HbA1c levels, FINDRISC, Type 2 diabetes

## Abstract

**Background:**

Prevalence of prediabetes and type 2 diabetes mellitus (T2DM) is increasing worldwide. The objective of this study was to determine the proportion of people in Northern Iceland with prediabetes, at risk of developing T2DM or with manifest undiagnosed T2DM, as this information is lacking in Iceland.

**Methods:**

A cross-sectional study. Clients of the three largest primary health care centres in the Health Care Institution of North Iceland (HSN) were invited to participate if fulfilling the following inclusion criteria: a) aged between 18 and 75 years, b) not diagnosed with diabetes, c) speaking and understanding Icelandic or English fluently and d) living in the included service area.

Data collection took place via face-to-face interviews between 1 March 2020 and 15 May 2021. Participation included answering the Finnish Diabetes Risk Score (FINDRISC), measuring the HbA1c levels and background information.

**Results:**

Of the 220 participants, 65.9% were women. The mean age was 52.1 years (SD ± 14.1) and FINDRISC scores were as follows: 47.3% scored ≤8 points, 37.2% scored between 9 and 14 points, and 15.5% scored between 15 and 26 points. The mean HbA1c levels in mmol/mol, were 35.5 (SD ± 3.9) for men and 34.4 (SD ± 3.4) for women, ranging from 24 to 47. Body mass index ≥30 kg/m^2^ was found in 32% of men and 35.9% of women. Prevalence of prediabetes in this cohort was 13.2%. None of the participants had undiagnosed T2DM. Best sensitivity and specificity for finding prediabetes was by using cut-off points of ≥11 on FINDRISC, which gave a ROC curve of 0.814.

**Conclusions:**

The FINDRISC is a non-invasive and easily applied screening instrument for prediabetes. Used in advance of other more expensive and invasive testing, it can enable earlier intervention by assisting decision making, health promotion actions and prevention of the disease burden within primary health care.

**Trial registration:**

This study is a pre-phase of the registered study “Effectiveness of Nurse-coordinated Follow up Program in Primary Care for People at risk of T2DM” at www.ClinicalTrials.gov (NCT01688359). Registered 30 December 2020.

## Introduction

Type 2 diabetes mellitus (T2DM) is among the fastest growing challenges to good health [1, 2]. Prevalence has tripled worldwide the last twenty years [3]. Today, over 463 million people are estimated to be living with diabetes [4], by 2030, 478 million over 18 years will have the disease [3], and 700 million by 2045 [5].

T2DM is the most common type of diabetes, often characterized by a silent onset of increased insulin resistance or lack of insulin production [4]. T2DM accounts for over 90% of diabetes cases [4, 6]. There is often a time lag between onset and formal diagnosis [7]. In the United States (U.S.), average interval between the onset of T2DM and diagnosis was found to be 7 years [8], and it is claimed that up to 30% of people with T2DM are undiagnosed [8]. The International Diabetes Federation (IDF) estimates that up to half of cases (49.7%) may not know that they have the disease [3].

A study on prevalence of diabetes by the Icelandic Heart Association from 1967 to 2007 found one out of three people with T2DM were unaware of their condition, when measuring fasting blood glucose [9, 10]. This included data from several population studies: 17,757 people aged 45-64 years for T2DM and three additional studies of 20,519 people for BMI [9, 10]. Unawareness of T2DM may increase the risk of chronic diabetes complications [11–13]. Those complications, for example, coronary arterial disease, renal failure, and blindness, may reduce the health-related quality of life for the individual [14], while society pays a high price due to impaired working capacity and high medical costs [3, 15]. If untreated, early onset T2DM may confer a higher lifetime cardiovascular and premature death risk compared to later onset of the disease [16]. The development of T2DM is the result of interaction between multiple environmental, socioeconomic, and genetically driven causes, changes toward a more sedentary lifestyle and less healthy diet choices [3, 17–19].

A strong connection has been found between T2DM and obesity [20, 21] Research indicates that obesity affects insulin resistance, and certain metabolites may show connection to both T2DM and obesity [22]. Until 1981, the mean BMI in Iceland was under 25 in all age groups (25–75 years) [9]. Between 1968 and 2012, the BMI of those aged between 50 and 69 years increased by 11% for men and 8% for women [9]. Data from the Icelandic Heart Association survey of 17,757 Icelandic people between 1967 and 2007 showed that four out of five people with T2DM were obese [9]. The prevalence of T2DM in Iceland more than doubled in both men and women between 2005 and 2018 [23].

Prediabetes is a state of elevated glucose levels in the blood, without reaching the diagnostic blood glucose levels of T2DM [2]. The prevalence of prediabetes has risen in England, for example, from 11.6% in 2003 to 35.3% in 2011 [24]. In the U.S., biomarkers of prediabetes are found in about 25% of young adults [25]. A recent meta-analysis by Barry et al., showed that when combining data from five studies in middle-aged participants, that used both the WHO and ADA definition, the overall overlap prevalence of prediabetes was 27% [26]. The number of people with prediabetes in Iceland is unknown. Earlier Icelandic studies on the prevalence of T2DM have mostly used data from the capital area [23].

Emerging evidence from well-designed randomised controlled trials (RCTs) has shown that lifestyle intervention with or/without pharmacological treatment can prevent or delay the onset of T2DM among people at the prediabetes stage [9, 18, 27]. Finding those at risk earlier may be a way to reduce the disease burden, complications and cost for both individuals and society [4, 11, 28]. The American Diabetes Association (ADA) recommends screening people at risk of developing T2DM at least every third year and yearly after reaching 45 years [20]. The availability of suitable tools in the primary health care (PHC) for early identification is one of the biggest challenges in promoting health and disease prevention [11, 29]. A non-invasive screening test for risk assessment, such as The Finnish Diabetes Risk Score (FINDRISC), may be a useful tool to find undiagnosed and those at risk for developing T2DM [5, 30].

Accordingly, the objective of this study was to identify people with prediabetes or undiagnosed T2DM in Northern Iceland and to determine the usability of FINDRISC as a screening tool in Icelandic PHC, by comparing the results of HbA1c measurement to the FINDRISC score by calculating sensitivity and specificity of FINDRISC.

## Methods

### Study design and settings

This was a cross-sectional study conducted within the Health Institution of North Iceland (HSN). The HSN runs 18 PHC centres and 4 small hospitals in the Northern Iceland, serving around 36,000 inhabitants or around 10.4% of the total population of 368,792 inhabitants in Iceland (as of 1 January 2021) [31]. The study’s settings were the three largest PHC centres in the HSN. Located in Saudarkrokur, Akureyri and Husavik, they serve over half of the total population in the Northern part of Iceland. Primary health care in Iceland is funded by the state, although non-pensioners pay a small fee to visit the doctor or nurse (this was approximately 3.90 US$/3.60 € in 2021). A routine health check-up with a doctor or a nurse is not standard in the PHC system, except for maternity care and infant protection programmes. During the Covid-19 pandemic, only those with acute medical needs were allowed into PHC centres.

### Study population

All inhabitants living in the three study areas were eligible for the study if they were aged between 18 and 75 years, had not been diagnosed with diabetes, and spoke and understood Icelandic or English fluently. Around 17,600 inhabitants living in the service areas of the three PHCs fulfilled the age criteria. Exclusion criteria was diagnosed with T1DM or with active treatment of T2DM.

### Data collection

All data were collected in one-to-one interviews by the first author (EA). Data collection took place between February 2020 and May 2021. The original plan of approaching participants at the three participating PHC centres had to be changed as a result of strict restrictions on all unnecessary visits to the PCH centres due to the Covid-19 pandemic. Therefore, the first 101 participants were recruited via introduction letters handed out by a receptionist when they came for a visit to the PHC centre between February and May 2020. The remaining 119 participants were recruited via flyers and advertisements in local papers from January to May 2021.

The letters, advertisements and flyers gave information about the study and contact information for the first author. Participants who were approached at the PCH centres met the first author for data collection after their pre-booked appointment with their nurse or doctor at the PHC centre. However, participants recruited through advertisements or flyers, contacted the first author via phone, SMS, or e-mail. The first author then briefed them about the study and sent them in advance a copy of the introduction letter via e-mail. If that was not possible, the introduction letter was handed to participants before data collection at the prearranged time. Those who did not show up at the prearranged time for data collection were offered new appointments twice more but were then seen as unwilling to participate. Data from the participants were collected at the local PHC centres in Húsavík and Sauðárkrókur. In Akureyri, data were collected at the research centre.

### Biological and demographical measurements

Biological measurements were conducted by the first author and information on the results was given to each participant at the end of the data collection interview. BMI was calculated by weighing participants on a digital scale, in light clothing without shoes, to the nearest 100 g, and measuring their height to the nearest 0.1 cm with a portable measuring tape. Waist circumference was measured 2 cm above navel, with measuring tape of 1,5 m or 3 m capacity, constancy of both tapes was measured regularly. The HbA1c levels were analysed by a DCA Vantage® analyser, that is clinically proven correlating with lab methods [32], using capillary blood samples, and the device was calibrated according to instructions.

Participants answered the FINDRISC [33, 34] and gave additional background information on age, gender, educational level, occupation and living status using a laptop computer. They were offered paper as an alternative way to answer the questions and five participants requested this option. Another five participants informed the researcher that they had reading difficulties. For these participants, the questions were read out aloud, but the participants marked down their answers.

### Definition of diabetes, prediabetes and the HbA1c measurements

Prediabetes is manifested and diagnosed by impaired fasting glucose (IFG) of 5.6 to 6.9 mmol/L, an impaired two-hour plasma glucose with 75 g oral glucose tolerance test (OGTT) results of 7.8 to 11.0 mmol/L or, as used here, elevated levels of glycated haemoglobin (HbA1c) [35]. HbA1c is a test of glycated haemoglobin that is approved by the World Health Organization (WHO) as a diagnostic test of diabetes [1, 2]. The ADA definition of prediabetes was used in this study [20]. As participants with HbA1c levels between 39 and 47 mmol/mol (5.7–6.4%) were classified as having prediabetes but, those with HbA1c levels of ≥48 mmol/mol (≥6,5%) as having T2DM [17, 35].

### Instrument

FINDRISC is a commonly used instrument, designed to screen for unidentified diabetes and to identify people with an elevated risk of developing T2DM within the next 10 years [19, 33]. It was developed in Finland between 1987 and 1992 and is claimed to be the most frequently used diabetes risk screening instrument worldwide [36].

The FINDRISC instrument includes eight questions on age, gender, BMI, waist circumference, daily physical activity, consumption of fruit and vegetables, history of high blood pressure, pervious history of diabetes and family history of diabetes [33, 34]. Scores range from 0 to 26 points. A higher score represents a higher risk of developing the disease within the next 10 years. A score of under 7 points is regarded as low risk (1:100), 7–11 points represent a slightly elevated risk (1:25), 12–14 points indicate a moderate risk (1:6), over 15 points a high risk (1:3) and over 20 points (1:2) a very high risk [34].

FINDRISC is easy to use, non-invasive, inexpensive, includes modifiable risk factors such as diet, physical activity, and body weight [19]. It has been validated in several populations [37, 38], as well as in Iceland [39].

### Statistical analysis

Descriptive statistics were used for calculating means, standard deviations, and ranges to describe continuous variables. For categorical variables, counts and proportions were used. Comparison of the sample characteristics was preformed using t-tests for continuous variables and chi-square tests for categorical variables. BMI calculations were done for each participant using the Microsoft Excel calculator and correlation for BMI and HbA1c results.

Chi-square tests were used to calculate the sensitivity and specificity of different FINDRISC points using the HbA1c results of all participants. Participants were divided into two groups: one with normal Hba1c levels and one with prediabetes levels of HbA1c.

When analysing the utility of FINDRISC to predict the future risk of developing T2DM or prediabetes in this cohort the receiver-operating characteristic (ROC) curves were constructed, by using different cut-off points of FINDRISC. Area under the ROC curve or AUC presents how well test, here FINDRISC, is capable to differentiate between cases, here prediabetes or not. An AUC of 1.0 indicates perfect test accuracy with neither false positives nor false negatives. However, an AUC curve of 0.5 indicates that the results are no better than chance. With FINDRISC, the best cut-off points to identify prediabetes were those with the shortest distance to the upper left corner of the ROC curve [36].

The dataset was analysed with IBM SPSS statistics 27. Missing data, if applicable were excluded listwise. Significant statistical difference two tailed was set at *p* ≤ 0.05.

### Ethical considerations

The Icelandic National Bioethics Committee (VSN) (VSN-19-080-S1 approved 14/05/2019 and VSN-19-080-V1 approved 14/01/2020), also the Senior management of HSN, approved the research. All participants signed an informed consent form before participating in the study.

## Results

### Participants and background information

The background information of participants is described in Table 1. Mean age was 52.1 (SD ± 14.1, range from 18 to 75) years, and women were 65.9%. Almost half of the participants had completed a university degree.

**Table 1:** Background information by gender, age, living status, educational level, occupational status, and residency

	Total (***n*** = 220)	Men (***n*** = 75)	Women (***n*** = 145)
**Mean age** in years (18–75 years)	52.1 (SD ± 14.1)	52.7 (SD ± 13.7)	51.8 (SD ± 14.3)
**Living status**	**n (%)**	**n (%)**	**n (%)**
Living alone	17 (7.7)	6 (8.0)	11 (7.6)
Living with one other person	99 (45.0)	36 (48.0)	63 (43.4)
Living with two or more persons	102 (46.4)	32 (42.7)	70 (48.3)
Living status missing	2 (0.9)	1 (1.3)	1 (0.7)
**Educational level**	**n (%)**	**n (%)**	**n (%)**
Elementary school/junior high school or equal	52 (23.6)	15 (20.0)	37 (25.5)
Upper secondary school/vocational training/senior high school or equal	65 (29.5)	30 (40.0)	35 (24.1)
University degree	101 (45.9)	29 (38.7)	72 (49.7)
Educational level missing	2 (0.9)	1 (1.3)	1 (0.7)
**Occupational status**	**n (%)**	**n (%)**	**n (%)**
Working partly or full time	174 (79.1)	61 (81.3)	113 (77.9)
Unemployed	6 (2,7)	2 (2.7)	4 (2,8)
Pensioner (disabled/elderly pensioner)	34 (15.5)	12 (16.0)	22 (15.2)
Other	6 (2.7)	0 (0.0)	6 (4.1)
**PHC centre**	**n (%)**	**n (%)**	**n (%)**
Akureyri	109 (49.5)	39 (35.8)	70 (64,2)
Husavik	40 (18.2)	13 (32.5)	27 (67.5)
Saudarkrokur	71 (32.3)	23 (32.4)	48 (67.6)

### HbA1c levels

The HbA1c levels are shown in Table 2. Levels ranged from 24 to 47 mmol/mol. None was found with HbA1c at diabetes level. A total of 29 participants (13.2%) were measured with the ADA definition of prediabetes at 39–47 mmol/mol, of these, 14 were men and 15 women. The normal HbA1c group had mean levels of 33.8 mmol/mol (SD ± 2.7). Contrast showed for the people with prediabetes HbA1c level had mean levels of 41.5 mmol/mol (SD ± 2.2). No significant difference in HbA1c was identified between genders, although a tendency toward higher HbA1c levels in men was observed (*p* = 0.056).

**Table 2:** HbA1c level of the participants according to gender, number and (percentage)

HbA1c Level mmol/mol	Total n (%)	Men n (%)	Women n (%)
24–38	191 (86.8)	61 (81.3)	130 (89.7)
39	5 (2.3)	2 (2.7)	3 (2.1)
40–41	12 (5.5)	9 (12.0)	3 (2.1)
42–47	12 (5.5)	3 (4.0)	9 (6.2)
Lowest level		24	25
Mean		35.48 (SD ± 3.92)	34.44 (SD ± 3.55)

### Body Mass Index

BMI ranged from 18.5 to 48.2 kg/m^2^, with a mean 28.8 (SD ±5.4) and median 27.7 kg/m^2^. Results showed that 39.1% were overweight (25–30 kg/m^2^) and obesity (≥ 30 kg/m^2^) was found in 34.5% of participants, 5% had severe obesity of BMI over 40 kg/m^2^. No significant difference in BMI was found regarding to gender (*p* = 0.982). The BMI scores among the 29 participants with prediabetes ranged between a minimum of 24.4 kg/m^2^ and a maximum of 48.2 kg/m^e^ with a mean of 32.3 kg/m^2^ (SD ± 5.7). BMI scores among the 191 participants who did not have elevated HbA1c levels ranged between 18.5 kg/m^2^ and 43.6 with a mean of 28.3 kg/m^2^ (SD ± 5.2). There was a significant positive correlation between BMI and HbA1c levels for all participants: r _(218)_ = 0.146, *p* = 0.044.

### FINDRISC scores

The characteristics of FINDRISC according to gender are reported in Table 3. One in five (21.4%) had first degree relatives with T2DM. A significant difference between gender was only found in the daily consumption of fruit and vegetables, in favour of women (*p* = 0.004). There was no significant gender difference in FINDRISC score, with a mean score for men of 9.0 (SD ± 5.3) and for women of 9.7 (SD ± 4.9), *p* = 0.332. However, there was a significant difference between the normal HbA1c group (*n* = 191) and the prediabetes group (*n* = 29) with FINDRISC scores of 8.6 (SD ± 4.5, range 0–22) points and 14.7 (SD ± 5.2, range 3–24) points, respectively (*p* < 0.001).

**Table 3:** Overall FINDRISC scoring and according to gender

	Scoring Points (P)	Overall (***n*** = 220)	Gender		***P***
			Men (***n*** = 75)	Women (***n*** = 145)	
**Age**		n (%)	n (%)	n (%)	.982
18–44 years	(0 P)	56 (25.5)	18 (24.1)	38 (26.2)	
45–54 years	(2 P)	56 (25.5)	19 (25.3)	37 (25.5)	
55–64 years	(3 P)	55 (25.0)	19 (25.3)	36 (24.8)	
65 years and older	(4 P)	53 (24.1)	19 (25.3)	34 (23.5)	
**BMI**					.838
< 25 kg/m^2^	(0 P)	58 (26.4)	21 (28.0)	37 (25.5)	
25–30 kg/m^2^	(1 P)	86 (39.1)	30 (40.0)	56 (38.6)	
≥ 30 kg/m^2^	(3 P)	76 (34.5)	24 (32.0)	52 (35.9)	
**Waist circumference (2 cm above navel)**					
Men < 94 cm/Women < 80 cm	(0 P)	63 (28.6)	30 (40.0)	33 (22.8)	
Men 94–102 cm/Women 80–88 cm	(3 P)	49 (22.3)	14 (18.7)	35 (24.1)	
Men > 102 cm/Women > 88 cm	(4 P)	108 (49.1)	31 (41.3)	77 (53.1)	
**Physically active ≥ 30 min/daily**					.672
Yes	(0 P)	203 (92.3)	70 (93.3)	133 (91.7)	
No	(2 P)	17 (7.7)	5 (6.7)	12 (8.3)	
**Fruit & vegetables daily**					.004^a^
Every day	(0 P)	140 (63.6)	38 (50.7)	102 (70.3)	
Not every day	(1 P)	80 (36.4)	37 (49.3)	43 (29.7)	
**Use of blood pressure medicine**					.085
No	(0 P)	165 (75.0)	51 (68.0)	114 (78.6)	
Yes	(2 P)	55 (25.0)	24 (32.0)	31 (21.4)	
**History of high blood glucose (including gestation diabetes)**					.130
No	(0 P)	192 (87.3)	69 (92.0)	123 (84.8)	
Yes	(5 P)	28 (12.7)	6 (8.0)	22 (15.2)	
**Family history of diabetes**					.743
Non	(0 P)	138 (62.7)	48 (64.0)	90 (62.1)	
First degree relatives	(3 P)	47 (21.4)	17 (22.7)	30 (20.7)	
Second degree relatives	(5 P)	35 (15.9)	10 (13.3)	25 (17.3)	
**FINDRISC SCORE**					.733
	≤ 8	104 (47.3)	37 (49.4)	67 (46.2)	
	9–11	43 (19.5)	16 (21.3)	27 (18.6)	
	12–14	39 (17.7)	13 (17.3)	26 (17.9)	
	15–20	27 (12.3)	6 (8.0)	21 (14.5)	
	21–26	7 (3.2)	3 (4.0)	4 (2.8)	

^a^Significant at the < 0.05 level

### Sensitivity and specificity

Table 4 shows the results of sensitivity and specificity calculations for different cut-off points of FINDRISC from 9 to15 points, with FINDRISC set as an outcome in the sensitivity and specificity calculation using the HbA1c level of prediabetes/not prediabetes as criteria. Best sensitivity (93.1%) of FINDRISC was found at ≥9 points, finding 27 of 29 participants with HbA1c levels at prediabetes value, but specificity was low only 53.4%. Using cut-off value of ≥11 or ≥ 12 points gave a little lower sensitivity (79.3 and 75.9% respectively) but better specificity (67 and 73.3% respectively) with fewer false positive. Using ≥15 points on FINDRISC resulted in a sensitivity of only 41.4%, missing more than half of those with a prediabetes HbA1c value, though the specificity was high (88.5%).

**Table 4:** Sensitivity and specificity of FINDRISC in predicting prediabetes risk, using cut-off-points at: ≥9, ≥10, ≥11, ≥12, ≥13, ≥14 or ≥ 15 points and HbA1c level 39 -47 mmol/mol as value for prediabetes

FINDRISC SCORE	Sensitivity true positive	Specificity true negative
≥9 points	93.1%	53.4%
≥10 points	79.3%	67.0%
≥11 points	79.3%	67.0%
≥12 points	75.9%	73.3%
≥13 points	69.0%	81.2%
≥14 points	55.2%	83.8%
≥15 points	41.4%	88.5%

By using ≥11 points as cut-off value of FINDRISC, calculations of the ROC curve showed area under the curve (AUC) to be 0.814, with 95% confidence interval (CI), lower bound of 0.733, an upper bound of 0.895 and standard error of 0.041. The result indicated that the best accuracy of finding people with prediabetes in this study was a FINDRISC score of ≥11 points, which gave the value closest to the upper left corner of the ROC curve.

## Discussion

This is the first study of prediabetes and undiagnosed T2DM prevalence in Iceland using both measurements of HbA1c level and FINDRISC score. Although none of the participants in our study was found with undiagnosed diabetes, 13.2% had biomarkers of prediabetes. Our sample was similar to the general population in the three study areas according to age and educational level, but this was not the case with the gender ratio. In our study, 65.9% of the participants were women, whereas the ratio in the general population is even [40]. The fact that fewer men than women participated in the study may partly explain why no participants were found with undiagnosed T2DM as more men than women are diagnosed with T2DM [23].

If the results are representative for the prevalence of prediabetes in the Northern Iceland areas of the study, this gives us a cautious indication that approximately 2300 of 17,600 inhabitants, within the age limits of the study and more men than women, may unknowingly have biomarkers of prediabetes. This is a cautious indication as the calculation of the confidence level of the sample size gave an 85% confidence level with a 5% margin of error, which indicates that up to 35,000 of approximately 266,000 inhabitants in Iceland, aged between 18 and 75 years as of 1 January 2021 [31], may show biomarkers of prediabetes. That is nearly equal to the total population of North Iceland. As this is the first study to assess prediabetes in Iceland, we are unable to compare our results with other results or other areas in Iceland. Here, the prevalence of prediabetes was lower than reported in a cross-sectional study in the Faroe Islands conducted in the years 2011-12, using HbA1c measurements followed by the non-fasting and fasting glucose and oral glucose tolerance test (OCTT). That study found that the prevalence of prediabetes was 22.3% in the age group of 44–77 years, with prevalence increasing with age [41]. The NANES cross-sectional survey estimated that the prevalence of prediabetes in the U.S. might be up to 38.6% in 2017 [42]. The fact that our sample included fewer men than in the general population in the area may partly explain why the prevalence of prediabetes was low. Also, that the participants were self-selected and had high educational level may have influenced the results. It should be kept in mind that the incidence of T2DM in Iceland is increasing. Research results using data on prescribed medicine for T2DM estimate that 10,600 individuals had T2DM in the year 2018 and that by 2040 up to 24,000 individuals may have the disease [43].

Obesity has been linked to an increased diabetes risk [21], and was found in 34.5% of the participants. That is much higher than earlier Icelandic results. The restriction on unnecessary visits to PHC centres during the Covid-19 pandemic may partly explain the high obesity figures. In 2007, obesity was found in 20% of Icelanders, whereas it was 27% in 2017, giving Iceland a ranking of number two in the prevalence of obesity within the OECD countries [43]. Earlier research indicates that women living outside the capital area in Iceland are more likely to be obese [44]. Public health care needs to find ways to reverse this trend by increasing awareness of the adverse consequences of obesity for the health of the population.

The results of our ROC curve calculations showed that the optimal cut-off point on FINDRISC was at ≥11 points, with AUC at 0.814, indicating the best sensitivity and specificity for predicting the risk of prediabetes in the sample. Our results are comparable to results from the HUNT study in Norway which suggests using ≥11 points on FINDRISC as the cut-off point for predicting risk of T2DM [45]. Other research suggests different cut-off points. Meijnikman et al. claim that a score of ≥13 points have the best sensitivity and specificity in overweight or obese people [36]. Using FINDRISC in a Lebanese University, Abdallah et al. found that the best cut-off score was ≥9.5 points for prediabetes and ≥ 10.5 points for undiagnosed T2DM [46]. The HUNT study and our study used a population-based sample to analyse the best cut-off score. In addition, the health care systems in the Nordic countries are similar [43]. However, validation of the best cut-off score for each population should be confirmed [36].

FINDRISC is non-invasive and easily applied instrument within PHC to identify those that may benefit from intervention for health promotion [38, 46]. The results showed higher mean scores on FINDRISC for the group with HbA1c levels of prediabetes compared to the group with normal HbA1c levels. But within the normal HbA1c group, FINDRISC scores of up to 22 points were found, indicating an up to 50% likelihood of developing T2DM in the next 10 years. It can therefore be recommended that health care providers in PHC use FINDRISC to screen for elevated risk of T2DM among their clients aged between 18 and 75 years. Clients who score ≥ 11 points should also be examined by measuring their HbA1c levels or blood glucose.

Those found with HbA1c levels of prediabetes should be offered person-centred intervention towards lifestyle change, led by PHC nurses, with the aim to lower diabetes risk for the individual. Results of randomised trials on the effects of nurse-led interventions to help people at risk of or with T2DM, to move towards better self-management and better control of HbA1c, show signs of continuing benefits after the intervention is over [18, 47].

WHO calls for preventive pathways to find people at early stages of non-communicable diseases like prediabetes is in T2DM [48]. Research shows that only part of people with prolonged stage of elevated glycemia or prediabetes progress to diabetic stage while others return to normal glycemia [49–51]. It has been reported that lifestyle changes may reduce the relative risk of progressing from prediabetes to diabetes by 40 to 70% [52]. Prior research indicates that physician’s attitude toward diabetes prevention may make an impact on screening for and treatment of prediabetes in PHC [53, 54]. It may not be customary practice to treat people actively where prediabetes is found [55]. However, screening to find those at risk offers grounds for intervention and health promotion as there may be time for patients to bring glycaemia levels back to normal [49–51]. Lifestyle changes that minimise the risk of further development of prediabetes are found to be cost-effective [20].

### Strength of the study

All participants were measured by the same person, minimising differences between measurements. The sample size is acceptable and representative according to the total population. This is the first study in Northern Iceland to measure the prevalence of prediabetes and undiagnosed diabetes.

### Limitations

Due to the Covid-19 epidemic, the recruitment of participants took longer than anticipated and the recruitment plan had to be changed. The participants were self-selected, not randomly assigned to the study. This may have affected the study sample as the educational level was high and more women than men participated, although the gender ratio is almost equal in the study population [40]. In addition, as the prevalence of prediabetes was found to be lower compared to research in other countries, there is a possibility that low prevalence here might have affected results of level of the FINDRISC score of ≥11 points as screening for prediabetes, therefore further research is needed.

FINDRISC has been criticised for not fully capturing the impact of gender and age. It may identify more women than men as high-risk individuals and therefore the cut-off points on FINDRISC may need to be adjusted for gender [56]. This criticism was not sustained in this study, but the uneven gender proportion may explain why no gender difference was found in the FINDRISC scores.

## Conclusion

Prevalence of overweight and obesity was high in this study. Despite the known increasing incidence of T2DM and association of overweight and higher risk of T2DM, this survey did not identify people with previously unknown T2DM. People found with biomarkers of prediabetes were fewer than expected in comparison to other countries. These favorable results may reflect the solid health care in Iceland. Also, plausible raising awareness of the importance of health promotion interventions within the Icelandic PHC.

FINDRISC is a short, non-invasive, easily applied instrument that this study found useful in screening within PHC for people at risk of prediabetes. The instrument can be used in advance of other more expensive and invasive testing for prediabetes or undiagnosed T2DM to enable earlier intervention by assisting decision making, health promotion actions toward prevention of T2DM.

### Key results

13.2% was found with prediabetes level of HbA1c, unaware of their condition.

FINDRISC is a non-invasive and an effective screening tool within the PHC to find people at prediabetes level, using score of ≥11 to set as consideration of further testing of HbA1c level or other more invasive screening of T2DM.

## Data Availability

The datasets used and/or analyzed during the current study available from the corresponding author on reasonable request.

## Abbreviations

ADA: American Diabetes Association
FINDRISC: Finnish Diabetes Risk Score
HbA1c: Glycated Haemoglobin
HSN: Health Institute of North Iceland
IDF: International Diabetes Federation
IFG: Impaired Fasting Glucose
OGTT: Oral Glucose Tolerance Test
PHC: Primary Health care
T2DM: Type two Diabetes mellitus
VSN: The Icelandic National Bioethics Committee

## References

[1] World Health Organization. Definition, diagnosis and classification of diabetes mellitus and its complications: Report of a WHO consultation part 1, diagnosis and classification of diabetes mellitus (no. WHO/NCD/NCS/99.2). 1999. Available from: https://apps.who.int/iris/handle/10665/66040.

[2] World Health Organization. Use of glycated haemoglobin (HbA1c) in diagnosis of diabetes mellitus: Abbreviated report of a WHO consultation (no. WHO/NMH/CHP/CPM/11.1). 2011. Available https://apps.who.int/iris/bitstream/handle/10665/70523/WHO_NMH_CHP_CPM_11.1_eng.pdf. 26158184

[3] Cho N, Shaw JE, Karuranga S (2018). IDF diabetes atlas: Global estimates of diabetes prevalence for 2017 and projections for 2045. Diabet Res Clin Pract..

[4] Gregg E, Buckley J, Ali M, Davies J, Flood D, Griffiths B, Lim LL, Manne-Goehler J, Pearson-Stuttard J, Shaw J. Improving health outcomes of people with diabetes mellitus: Target setting to reduce the global burden of diabetes mellitus by 2030. 2021. https://cdn.who.int/media/docs/default-source/searo/india/health-topic-pdf/noncommunicable-diseases/eb150---annex-2-(diabetes-targets)---fin.

[5] Saeedi P, Petersohn I, Salpea P (2019). IDF diabetes atlas committee global and regional diabetes prevalence estimates for 2019 and projections for 2030 and 2045: Results from the international diabetes federation diabetes atlas. Diabet Res Clin Pract.

[6] WHO.int. Diabetes. 2020. Available from: https://www.who.int/health-topics/diabetes#tab=tab_1f.

[7] Kong AP, Luk AO, Chan JC (2016). Detecting people at high risk of type 2 diabetes-how do we find them and who should be treated?. Best Pract Res Clin Endocrin Meta.

[8] Zhang Y, Hu G, Zhang L, Mayo R, Chen L (2015). A novel testing model for opportunistic screening of pre-diabetes and diabetes among US adults. PLoS One.

[9] Thórsson B, Aspelund T, Harris TB, Launer LJ, Gudnason V (2009). Trends in body weight and diabetes in forty years in Iceland. Icelandic Med J..

[10] Thórsson B, Gudmundsson EF, Sigurdsson G, Aspelund T, Gudnason V. Prevalence and incidence of type 2 diabetes in Iceland 2005-2018. Icelandic Med J. 2021;107(5):227-33. 10.17992/lbl.2021.05.634 33904831

[11] World Health Organization (2021). Primary health care on the road to universal health coverage: 2019 global monitoring report.

[12] Fox CS, Coady S, Sorlie PD (2007). Increasing cardiovascular disease burden due to diabetes mellitus: The Framingham heart study. Circulation..

[13] Hanssen KF, Bangstad H, Brinchmann-Hansen O, Dahl-Jørgensen K (1992). Blood glucose control and diabetic microvascular complications: Long-term effects of near-normoglycaemia. Diabet Med.

[14] Trikkalinou A, Papazafiropoulou AK, Melidonis A (2017). Type 2 diabetes and quality of life. World J Diabet.

[15] Bähler C, Huber CA, Brüngger B, Reich O (2015). Multimorbidity, health care utilization and costs in an elderly community-dwelling population: A claims data based observational study. BMC Health Serv Res.

[16] Steinarsson AO, Rawshani A, Gudbjornsdottir S, Franzén S, Svensson A, Sattar N (2018). Short-term progression of cardiometabolic risk factors in relation to age at type 2 diabetes diagnosis: A longitudinal observational study of 100,606 individuals from the Swedish national diabetes register. Diabetologia..

[17] Boyko EJ, Karuranga S, Magliano DJ, Saeedi P, Sun H. IDF diabetes atlas 10 ed. 2019. Accessed 2 Aug 2021. https://diabetesatlas.org/idfawp/resource-files/2021/07/IDF_Atlas_10th_Edition_2021.pdf.

[18] Diabetes Prevention Program Research Group, (DPPRG) (2015). Long-term effects of lifestyle intervention or metformin on diabetes development and microvascular complications over 15-year follow-up: The diabetes prevention program outcomes study. Lancet Diabet Endocrin.

[19] Lindström J, Peltonen M, Eriksson JG (2008). Determinants for the effectiveness of lifestyle intervention in the Finnish diabetes prevention study. Diabetes Care.

[20] American Diabetes Association (ADA). Standards of medical care in Diabetes—2022 abridged for primary care providers. Clin Diabet. 2021. 10.2337/cd22-as01. 10.2337/cd22-as01 PMC8865785 35221470

[21] Schnurr TM, Jakupović H, Carrasquilla GD (2020). Obesity, unfavorable lifestyle and genetic risk of type 2 diabetes: A case-cohort study. Diabetologia..

[22] Park S, Sadanala KC, Kim E (2015). A metabolomic approach to understanding the metabolic link between obesity and diabetes. Mol Cells.

[23] Andersen K, Aspelund T, Gudmundsson EF (2017). Five decades of coronary artery disease in Iceland. Data from the Icelandic heart association. Icelandic Med J..

[24] Mainous AG, Tanner RJ, Baker R, Zayas CE, Harle CA (2014). Prevalence of prediabetes in England from 2003 to 2011: Population-based, cross-sectional study. BMJ Open.

[25] Andes LJ, Cheng YJ, Rolka DB, Gregg EW, Imperatore G (2020). Prevalence of prediabetes among adolescents and young adults in the United States, 2005-2016. JAMA Ped.

[26] Barry E, Roberts S, Oke J, Vijayaraghavan S, Normansell R, Greenhalgh T (2017). Efficacy and effectiveness of screen and treat policies in prevention of type 2 diabetes: Systematic review and meta-analysis of screening tests and interventions. BMJ..

[27] Diabetes Prevention Program Research Group (DPPRG) (2002). Reduction in the incidence of type 2 diabetes with lifestyle intervention or metformin. N Engl J Med.

[28] Wändell PE (2005). Quality of life of patients with diabetes mellitus an overview of research in primary health care in the Nordic countries. Scand J Prim Health Care.

[29] Lee A, Kiyu A, Milman HM, Jimenez J (2007). Improving health and building human capital through an effective primary care system. J Urban Health.

[30] Guo F, Moellering DR, Garvey WT (2014). Use of HbA1c for diagnoses of diabetes and prediabetes: Comparison with diagnoses based on fasting and 2-hr glucose values and effects of gender, race, and age. Met Syndrome Related Dis.

[31] Statistic Iceland. Population by localities, sex and age 1 January 2001-2021. 2021. https://px.hagstofa.is/pxen/pxweb/en/Ibuar/Ibuar__mannfjoldi__2_byggdir__Byggdakjarnar/MAN030101.px.

[32] Weykamp C, Siebelder C (2018). Evaluation of performance of laboratories and manufacturers within the framework of the IFCC model for quality targets of HbA1c. J Diabet Sci Tech.

[33] Lindström J, Tuomilehto J (2003). The diabetes risk score: A practical tool to predict type 2 diabetes risk. Diabetes Care.

[34] Finnish Diabetes Association. DEHKO – development programme for the prevention and care of diabetes in Finland 2000–2010. In: Etu-Seppälä L, IIanne-Parikka P, Haapa E, editors. Progamme for the prevention of type 2 diabetes in Finland 2003–2010: DEHKO Finnish Diabetes Association; 2003. Available from: https://www.diabetes.fi/files/200/Development_Programme_for_the_Prevention_and_Care_of_Diabetes_2000_2010_pdf_910_kB.pdf.

[35] American Diabetes Association (2021). Addendum. 2. classification and diagnosis of diabetes: Standards of medical care in Diabetes—2021. Diabetes Care.

[36] Meijnikman AS, De Block C, Verrijken A, Mertens I, Corthouts B, Van Gaal LF (2016). Screening for type 2 diabetes mellitus in overweight and obese subjects made easy by the FINDRISC score. J Diabet Compl.

[37] Silvestre MP, Jiang Y, Volkova K, Chisholm H, Lee W, Poppitt SD (2017). Evaluating FINDRISC as a screening tool for type 2 diabetes among overweight adults in the PREVIEW: NZ cohort. Prim Care Diabet.

[38] Gabriel R, Acosta T, Florez K (2021). Validation of the Finnish type 2 diabetes risk score (FINDRISC) with the OGTT in health care practices in Europe. Diabet Res Clin Pract..

[39] Ingvadottir JM, Sigurdardottir AK. Using FINDRISC to evaluate risk of T2DM in Iceland: Quantitative research (notkun FINDRISK-matstaekisins til ad meta haettu á sykursyki af tegund 2: Megindleg rannsokn). Icelandic J Nursing. 2017;93(1):60-66. ISSN 2298-7053.

[40] Skuladottir LK. East Northern Iceland: Status analysis 2019 (Nordurland eystra: Stodugreining 2019, (Icelandic)). https://www.byggdastofnun.is/static/files/Skyrslur/stgr19_20/nordurland-eystra-stodugreining-2019-2020-loka.pdf.

[41] Veyhe AS, Andreassen J, Halling J, Grandjean P, Petersen MS, Weihe P (2019). Prevalence of prediabetes and type 2 diabetes in two non-random populations aged 44–77 years in the Faroe Islands. J Clin Trans Endocrinol.

[42] Xia P, Tian Y, Geng T, et al. Trends in prevalence and awareness of prediabetes among adults in the US, 2005–2020. Diabetes Care. 2022. 10.2337/dc21-2100. 10.2337/dc21-2100 34872984

[43] OECD/European Observatory on Health Systems and Policies. Iceland: Country health profile 2021, state of health in the EU. 2021. https://eurohealthobservatory.who.int/publications/country-health-profiles.

[44] Guðjonsdottir H, Halldorsson TI, Gunnarsdottir I, Thorsdottir I, Thorgeirsdottir H, Steingrimsdottir L (2015). Urban-rural differences in diet, BMI and education of men and women in Iceland. Icelandic Med J..

[45] Jølle A, Midthjell K, Holmen J (2019). Validity of the FINDRISC as a prediction tool for diabetes in a contemporary Norwegian population: A 10-year follow-up of the HUNT study. BMJ Open Diabet Res Care..

[46] Abdallah M, Sharbaji S, Sharbaji M (2020). Diagnostic accuracy of the Finnish diabetes risk score for the prediction of undiagnosed type 2 diabetes, prediabetes, and metabolic syndrome in the Lebanese university. Diabetol Metab Syndr.

[47] Azami G, Soh KL, Sazlina SG (2018). Effect of a nurse-led diabetes self-management education program on glycosylated hemoglobin among adults with type 2 diabetes. J Diabet Res..

[48] World Health Organization (2019). No title. Non-communicable disease prevention and control: a guidance note for investment cases.

[49] Lee CMY, Colagiuri S, Woodward M (2019). Comparing different definitions of prediabetes with subsequent risk of diabetes: An individual participant data meta-analysis involving 76 513 individuals and 8208 cases of incident diabetes. BMJ Open Diabet Res Care..

[50] Richter B, Hemmingsen B, Metzendorf M, Takwoingi Y. Development of type 2 diabetes mellitus in people with intermediate hyperglycaemia. Coch Datab Syst Rev. 2018;10. 10.1002/14651858.CD012661.pub2 PMC6516891 30371961

[51] De Abreu L, Holloway KL, Kotowicz MA, Pasco JA. Dysglycaemia and other predictors for progression or regression from impaired fasting glucose to diabetes or normoglycaemia. J Diabet Res. 2015;2015. 10.1155/2015/373762 PMC4530268 26273669

[52] Tabák AG, Herder C, Rathmann W, Brunner EJ, Kivimäki M (2012). Prediabetes: A high-risk state for diabetes development. Lancet..

[53] Mainous AG, Tanner RJ, Baker R (2016). Prediabetes diagnosis and treatment in primary care. J Am Board Fam Med.

[54] Mainous AG, Tanner RJ, Scuderi CB, Porter M, Carek PJ (2016). Prediabetes screening and treatment in diabetes prevention: The impact of physician attitudes. J Am Board Fam Med.

[55] Mainous AG, Rooks BJ, Wright RU, Sumfest JM, Carek PJ (2022). Diabetes prevention in a US healthcare system: A portrait of missed opportunities. Am J Prev Med.

[56] Jølle A, Midthjell K, Holmen J (2016). Impact of sex and age on the performance of FINDRISC: The HUNT study in Norway. BMJ Open Diabet Res Care.

